# Rivaroxaban Treatment for Heparin-Induced Thrombocytopenia: A Case Report and a Review of the Current Experience

**DOI:** 10.1155/2020/8885256

**Published:** 2020-09-03

**Authors:** Mohamed Aon, Omar Al-Shammari

**Affiliations:** ^1^Department of Medicine, Jahra Hospital, Ministry of Health (HOM), Jahra city, Kuwait; ^2^Department of Medicine, Faculty of Medicine, Cairo University, Giza, Egypt

## Abstract

Heparin-induced thrombocytopenia is a life-threatening complication of exposure to heparin. Heparin-induced thrombocytopenia results from an autoantibody directed against platelet factor 4 in complex with heparin. Heparin-induced thrombocytopenia is traditionally treated with bivalirudin, argatroban, danaparoid, or fondaparinux. Recently, direct oral anticoagulants administration to treat heparin-induced thrombocytopenia has been reported. Direct oral anticoagulants do not cause platelet activation in the presence of heparin-platelet factor 4 antibodies, nor do they provoke autoantibody production. Direct oral anticoagulants offer advantages such as consistent and predictable anticoagulation, oral administration with good patient compliance, and a good safety profile. We report a case of heparin-induced thrombocytopenia with deep venous thrombosis successfully treated with rivaroxaban and review the current experience with rivaroxaban for the treatment of heparin-induced thrombocytopenia.

## 1. Introduction

Heparin-induced thrombocytopenia (HIT) is a clinical syndrome complicating exposure to heparin that occurs in a small percentage of patients. HIT is caused by antibodies to complexes of platelet factor 4 (PF4) and heparin that can cause thrombosis along with thrombocytopenia. HIT diagnosis rests on the presence of thrombocytopenia and/or thrombosis after heparin exposure while excluding other possible causes of thrombocytopenia [1]. HIT requires immediate treatment to reduce the risk of serious thrombosis. There are several nonheparin anticoagulants that can be used in the treatment of HIT, e.g., argatroban, bivalirudin, danaparoid, and fondaparinux. Accumulating evidence suggests that direct oral anticoagulants (DOACs) are effective in treating HIT [2].

In this article, we report a case of HIT complicated with deep venous thrombosis (DVT) successfully treated with rivaroxaban and review the accumulating experience with rivaroxaban for the treatment of HIT.

## 2. Case Presentation

**Figure 1:**
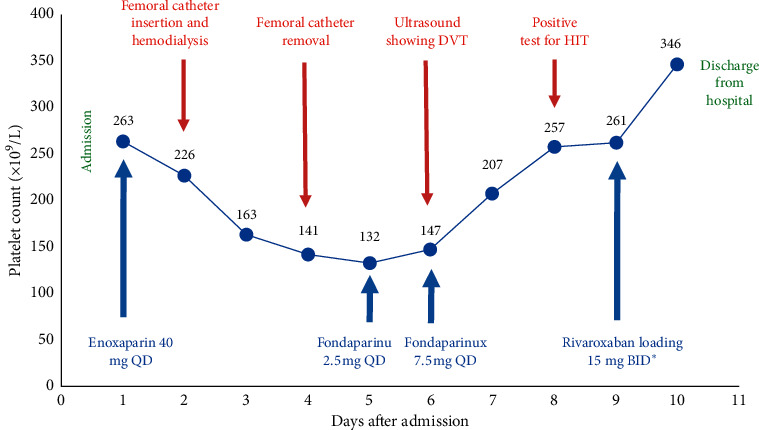
Platelet count charted against time and timeline of clinical events and medications used until day of discharge. QD, once daily; BID, twice per day; DVT, deep venous thrombosis; HIT, heparin-induced thrombocytopenia. ^*∗*^Loading for 21 days and then 20 mg QD to complete a total of three months.

A 25-years-old male presented to Jahra Hospital, Kuwait, with confusion after the ingestion of methanol. In the emergency room, he had high anion gap metabolic acidosis (pH 6.8, HCO_3_ 8 mmol/L, anion gap 31 mmol/L), elevated serum lactate (16.6 mmol/L), and hypoglycemia. Treatment was started with intravenous dextrose, thiamine, and sodium bicarbonate infusion. Due to persistent acidosis, dialysis was started through a right femoral catheter. After dialysis, his metabolic parameters improved, and the catheter was removed. His medication included enoxaparin 40 mg once daily for DVT prophylaxis, omeprazole, folic acid, and thiamine. On the 5th day of hospital stay, the patient's platelet count decreased (Figure 1), with normal white blood cells, hemoglobin, and coagulation profile, so his enoxaparin prophylaxis was substituted with fondaparinux 2.5 mg once daily. On the 6th day of hospital stay, he started to develop right-sided thigh pain and swelling. Urgent ultrasound examination of the deep venous system revealed right external iliac, femoral, and popliteal vein recent thrombosis. Fondaparinux was increased to a therapeutic dose according to his body weight (7.5 mg once daily) and HIT serology was sent. Over the next days, the patient's condition was improving, and blood examination showed recovery of platelet count (Figure 1). Heparin-induced anti-platelet antibodies were detected at 0.846 optical density (OD) units, and the confirmatory functional assay was positive. Given his clinical and laboratory improvement, we decided to start him on rivaroxaban. Loading dose 15 mg twice daily for 21 days was started and the patient was released to outpatient care. Follow-up in outpatient revealed continued clinical improvement. The treatment was well tolerated with no adverse effects and the dosing was switched to 20 mg once daily to complete a total of three months of treatment. Three months after the hospital discharge, the patient underwent outpatient examination that revealed a good overall condition with a normal platelet count and recanalization of the deep venous system on ultrasound examination. Rivaroxaban was stopped and the patient was instructed to avoid heparin for life.

## 3. Discussion

HIT is a clinical syndrome caused by antibodies to complexes of PF4 and heparin. This syndrome has also been called heparin-induced thrombocytopenia and thrombosis (HITT) because antibodies can cause thrombosis along with thrombocytopenia [1]. Antibody-coated platelets are removed by the reticuloendothelial system causing thrombocytopenia. Consumption of platelets at sites of thrombosis is another mechanism of thrombocytopenia in HIT [3]. HIT is a prothrombotic disorder and the primary mechanism of thrombosis is due to the binding of HIT antibodies to the platelet which elicits platelet activation and release of procoagulant microparticles. Platelet activation in HIT is also accompanied by endothelial cell activation and injury leading to increased tissue factor expression and thrombin generation [4]. Affected individuals have a 50% risk of developing new thromboembolic events which can involve both the arterial and the venous systems. The mortality rate is approximately 15%, and 10% of patients require amputations or suffer another major morbidity [5]. HIT is more common in surgical patients compared with medical patients and more common among females. Absolute risks of developing HIT after exposure to prophylactic unfractionated heparin is 2.6% versus 0.2% after low molecular weight heparin (LMWH) exposure [6]. HIT diagnosis rests on the presence of thrombocytopenia and/or thrombosis in temporal association with heparin exposure while excluding other causes of thrombocytopenia. If HIT is suspected, it is recommended to use the 4Ts score to estimate the probability of HIT [7]. If there is an intermediate to high probability of HIT (4Ts score ≥4), heparin should be discontinued and an immunoassay for HIT antibodies is obtained. If the immunoassay is positive, a functional assay is used to confirm the results. A functional assay may not be necessary for patients with a high probability score and a strongly positive immunoassay >2.0 OD units [8].

Given that platelet drop is 50% on the 5th day after LMWH exposure, associated with a new DVT, and no other causes for thrombocytopenia are present, our patient's 4Ts score is 7 points, i.e., a high probability of HIT. His immunoassay is positive (OD 0.846; OD values ≥0.400 are considered positive in our reference laboratory and the confirmatory test is positive).

Once HIT is diagnosed, heparin must be discontinued, and a nonheparin anticoagulant is used instead. The latest American Society of Hematology (ASH) HIT treatment guidelines endorse the use of either argatroban, bivalirudin, danaparoid, or fondaparinux. Because of their short duration of action, argatroban and bivalirudin are preferred in critically ill patients, patients with high bleeding risk, or patients who may need urgent procedures. However, they are expensive and require a hospital stay for parenteral administration and frequent monitoring. Fondaparinux is being increasingly used in clinically stable patients at average risk of bleeding. Danaparoid is no longer available in the United States and parts of Europe [8]. In our case, the patient's stable condition and the average risk of bleeding is the reason to choose fondaparinux as a nonheparin anticoagulant initially.

Accumulating evidence suggests that DOACs are effective in treating HIT, without stimulating HIT antibodies. DOACs are an attractive treatment option for HIT as they offer many potential benefits, including ease of administration, rapid onset of action, no required monitoring, the competitive cost to other anticoagulants, and efficacy during longer-term anticoagulation. However, the evidence for the efficacy of DOACs for HIT is only based on prospective cohort studies or retrospective case series while an RCT is lacking [2]. Ideally, an RCT demonstrating the efficacy of a DOAC in HIT is preferred before it becomes an approved treatment. However, the rarity of HIT and the challenges in diagnosis delay any trial recruitment.

With respect to the choice of a DOAC, most of the published experience in HIT is with rivaroxaban which is an oral direct factor Xa inhibitor. After carrying out a search on PubMed and MEDLINE database using the words “heparin-induced thrombocytopenia,” and “rivaroxaban,” 59 references were identified, dating from 2008 to 2020. Until February 2020, twenty-one articles in English contained reports of HIT cases treated with rivaroxaban: 1 prospective study, 5 retrospective cohort studies, and 14 case reports, totaling 65 patients. In the previously discussed literature, 61.5% of cases had HIT-related thrombosis, treatment with rivaroxaban was started while the patient was still thrombocytopenic (i.e., acute HIT) in 84.6% of cases, while it was initiated after platelet recovery (i.e., subacute HIT) in 15.4%. Out of the 65 cases, one (1.5%) had recurrent venous thromboembolism and one case (1.5%) experienced a moderate bleeding episode while receiving rivaroxaban (Table 1).

Rivaroxaban was the initial nonheparin anticoagulant selected to treat HIT (i.e., primary therapy of HIT) in 44.6% of HIT cases. Switch from another parenteral nonheparin anticoagulant to rivaroxaban therapy (i.e., secondary therapy of HIT) was noticed in 55.4% of cases. Half of the published cases used the standard dose regimen for venous thromboembolism: 15 mg twice daily for the initial 21 days followed by 20 mg once daily, while others used different dosing regimens (Table 1).

Of all the DOACs, rivaroxaban has the largest body of literature for its use in HIT and is the only DOAC that has been evaluated in a prospective study. While the current experience remains limited, it is suggestive of the potential role of rivaroxaban in HIT, which has led to its integration into the 2018 ASH guidelines with a conditional recommendation [8].

In our case, after the initial use of fondaparinux for 3 days, platelets recovered, and rivaroxaban was started before discharging the patient. We used the standard dose of rivaroxaban: 15 mg twice daily for the initial 21 days followed by 20 mg once daily for 3 months. After 3 months of treatment, rivaroxaban was stopped.

In conclusion, this case report demonstrates the effectiveness and safety of rivaroxaban in the treatment of HIT. Given the rarity of HIT itself, there are no consensus management guidelines for the treatment of HIT with rivaroxaban. More research is needed including encouraging physicians to present outcome data when using rivaroxaban, creating registries of HIT treatment with rivaroxaban and conducting studies that compare rivaroxaban and other nonheparin anticoagulants.

## Figures and Tables

**Table 1:** Cohort studies and case reports of rivaroxaban for the treatment of HIT.

Study reference	Number of patients	HITT^*∗*^	Rivaroxaban treatment for acute or subacute HIT^*∗*^	Primary or secondary treatment^*∗*^	Extension of thrombosis or major bleeding	Dose^*∗*^
[2, 9]	28	12/28	19/28 acute 9/28 subacute	13/28 primary 15/28 secondary	Yes^a,b^	20/28 standard dose 4/28 20 mg once daily 4/28 10 mg once daily
[10, 11]^c^	9	9/9	9/9 acute	9/9 primary	No	4/9 standard dose 5/9 10 mg once daily
[12]	9^d^	4/9	9/9 acute	9/9 secondary	No	9/9 20 mg once daily
[13]	3	2/3	3/3 acute	3/3 secondary	No	3/3 15 mg twice daily
[14]	2	2/2	2/2 acute	2/2 secondary	Yes^e^	2/2 Standard dose
[15]	1	No	Acute	Secondary	No	Not specified
[16]	1	Yes	Acute	Secondary	No	Standard dose
[17]	1	Yes	Acute	Primary	No	20 mg twice daily
[18]	1	No	Acute	Primary	No	10 mg once daily
[19]	1	Yes	Acute	Primary	No	Standard dose
[20]	1	Yes	Acute	Secondary	No	Standard dose
[21]	1	Yes	Acute^f^	Secondary	No	20 mg once daily
[22]	1	No	Acute	Primary	No	15 mg twice daily
[23]	1	Yes	Acute	Secondary	No	Not specified
[24, 25]^g^	1	Yes	Acute	Primary	No	Standard dose
[26]	1	Yes	Subacute	Primary	No	10 mg once daily
[27]	1	Yes	Acute	Primary	No	Not specified
[28]	1	Yes	Acute	Secondary	No	Standard dose
[29]	1	Yes	Acute	Secondary	No	Not specified
Total (%)	65	40/65 (61.5%)	55/65 acute (84.6%) 10/65 subacute (15.4%)	29/65 primary (44.6%) 36/65 secondary (55.4%)	1/65 extension of thrombosis (1.5%) 1/65 major bleeding (1.5%)	33/65 standard dose (51%) 28/65 other doses (43%) 4/65 not specified (6%)
Our case	1	Yes	Subacute	Secondary	No	Standard dose

^*∗*^See text for details. ^a^Possible extension of a catheter-related arm DVT with full recovery after catheter removal and despite the continuation of rivaroxaban. ^b^One rectal bleeding episode with a known gastric cancer occurred 9 days after discontinuing rivaroxaban and while receiving fondaparinux, so not included. ^c^The articles are combined because the 3 patients first reported by Ng et al. are also included among the 9 patients reported by Ong et al. ^d^Out of the 11 patients who received rivaroxaban, 2 tested negative for HIT antibodies and thus are excluded in this table. ^e^Moderate hemoptysis secondary to known squamous cell lung cancer. ^f^Platelets dropped <50% but did not drop below 150 × 10^9^/L. ^g^Articles in Spanish but data from Barlow et al.

## Data Availability

Access to data is restricted to keep patient's privacy. However, if deemed necessary, data will be provided by the corresponding author upon reasonable request after approval from the needed institutional committee.
